# Mind the (cultural competency) gap: bridging cultural barriers in physiotherapy practice – a quality improvement project

**DOI:** 10.1017/S1463423625100765

**Published:** 2026-02-02

**Authors:** Manish Gohil, Christine Comer, Paul M. Millington

**Affiliations:** 1 Senior Physiotherapist, Leeds Community Healthcare NHS Trust, Leeds, UK; 2 Assistant Professor, School of Allied Health Professions & Midwifery, Faculty of Health Studies, University of Bradford, Bradford, UK; 3 Clinical Research Fellow, Leeds Community Healthcare NHS Trust, Leeds, UK

**Keywords:** Cultural competency, educational programme, ethnicity, health inequalities, physiotherapy, self-assessment

## Abstract

**Introduction:**

The increasing diversity of the UK’s population, along with significant inequalities in health outcomes among diverse cultural and ethnic groups, highlights the critical need for healthcare providers, including physiotherapists, to deliver culturally competent care. Research demonstrates that cultural competency (CC) in healthcare can enhance patient outcomes, improve treatment adherence, and address health inequalities. This quality improvement project aimed to evaluate the impact of a brief training intervention on perceived CC among physiotherapists engaging in cross-cultural, clinical encounters.

**Methods:**

The design of this project was informed by principles of the Plan-Do-Study-Act (PDSA) cycle. Musculoskeletal physiotherapists voluntarily participated in this educational intervention by completing a CC e-learning course developed by Health Education England. A retrospective evaluation method was used, where participants rated their awareness, knowledge, and skills in providing care to diverse patient groups after completing the training as compared to before.

**Results:**

The pre-and post-training responses assessed three constructs of CC: awareness, knowledge, and skills. Significant increases were observed in the mean scores for each construct, as well as in the overall aggregate CC score, indicating measurable improvements following the training.

**Conclusions:**

Improvement in CC is viable and achievable through targeted training. CC is a lifelong, dynamic process that requires ongoing education. Therefore, further PDSA cycles are recommended with more advanced educational sessions. Additionally, future projects should assess the impact of enhanced CC on patient outcomes and experiences. Cultural competency must encompass cultural issues beyond racial and ethnic differences.

## Introduction

Many countries, including the United Kingdom, are experiencing an increase in cultural diversity because of migration and globalization (Hargreaves *et al*., 2006). The shifting demographics and evolving global economy, along with longstanding health inequalities among people from different cultural and ethnic backgrounds, highlight the critical need for healthcare providers such as physiotherapists to deliver culturally competent care (Campinha-Bacote, 2002; DiBiasio *et al*., 2023). Healthcare professionals are increasingly tasked with understanding and addressing the diverse cultural, linguistic, and health-related needs of these populations, each bringing unique health beliefs, behaviors, and risk factors (Alizadeh and Chavan, 2015; Robinson *et al*., 2021).

Health inequalities are closely linked to ethnicity. While people from diverse cultural and ethnic backgrounds tend to use healthcare services frequently, they report lower levels of satisfaction and face barriers to accessing high-quality care (O’Shaughnessy and Tilki, 2007). These challenges can lead to poorer health outcomes. To address this, healthcare providers must adopt culturally competent practices that respect and accommodate patients’ values, beliefs, and cultural practices (Beach *et al*., 2005; O’Shaughnessy and Tilki, 2007; Brooks *et al*., 2019).

Cultural competency (CC) is the process by which healthcare providers strive to work and communicate effectively with people from diverse backgrounds (Argyriadis *et al*., 2022). While the term *‘*competency*’* may suggest the achievement of a sufficient qualification, it is now accepted that CC is an ongoing process rather than an endpoint event (Alizadeh and Chavan, 2015; Fryer *et al*., 2020). By embedding CC within healthcare practice, clinical staff can improve the quality of patient interactions and outcomes (Govere and Govere, 2016; Henderson *et al*., 2018). Specifically, CC enhances treatment adherence and helps reduce health inequalities by fostering better understanding and communication between providers and patients (May and Potia, 2013).

The literature highlights several reasons why healthcare professionals may fail to meet the needs of people from ethnic minority groups, including a lack of understanding of cultural diversity, racism, racial stereotyping, insufficient knowledge, exclusivity, and ethnocentrism (Chevannes, 2002; Alizadeh and Chavan, 2015; Brooks *et al*., 2019). Increasing awareness of CC can help reduce these barriers, enabling patients from diverse cultural backgrounds to fully benefit from healthcare services (Chevannes, 2002; Priebe *et al*., 2011; Govere and Govere, 2016; Robinson *et al*., 2021).

The need for physiotherapists to practise CC in patient interactions is recognized globally. CC is considered central to overall professional competence and capability (CSP, 2012; Fryer *et al*., 2020; HCPC, 2023). However, evidence suggests that physiotherapists often feel inadequately trained to manage patients from diverse cultural and ethnic backgrounds (Black and Purnell, 2002; O’Shaughnessy and Tilki, 2007; May and Potia, 2013). This gap highlights the importance of targeted educational interventions to enhance CC among physiotherapists and support equitable, patient-centred care.

Our project was undertaken in a UK-based community musculoskeletal (MSK) physiotherapy service where nearly 20% patients referred for care identify as being from ethnic minority groups (local NHS Trust data). In a city where over 25% of the city’s population is from ethnically diverse communities (Office for National Statistics, 2023), CC is crucial in ensuring the equality and effectiveness of MSK care. Previous studies in different settings and populations have shown benefits from a range of CC training methods including experiential learning, self-reflection, workshops, and online and in-person instruction (Khanna *et al*., 2009; Oikarainen *et al*., 2019; Fryer *et al*., 2020; Slobodin *et al*., 2021). The aim of our project was to determine if completing an online CC training intervention would lead to a measurable improvement in MSK physiotherapists’ perceived awareness, knowledge, and skills in providing care to patients from diverse cultural and ethnic minority backgrounds.

## Methods

The model guiding this project was the Plan-Do-Study-Act (PDSA) cycle, a widely recognized framework for healthcare improvement (Knudsen *et al*., 2019). During the planning phase, focus group discussions were held with MSK physiotherapists to explore their experiences, perceived barriers, and priorities in delivering culturally competent care. These discussions also confirmed anecdotal reports of limited confidence in this area of practice.

Findings from the focus groups informed key aspects of project design, including participant selection, training setting, and timing of the intervention. Practical considerations, such as whether training should be delivered virtually or in-person, and whether materials should be electronic or written, were also explored. On this basis, a pre-existing online CC training programme was selected for implementation.

### Participants

Participants were recruited from the team of total 69 registered MSK physiotherapists at Leeds Community Healthcare NHS Trust, who care for diverse patient groups and had not previously received CC training. All members of the team were invited to participate in the project through an email and verbal promotion.

Prior to participation in the project, all potential participants were sent an email providing relevant information about the project for them to read and consider before deciding whether or not to participate. This included information about what is CC, the reason for the project, and information about the retrospective post-then-pre-evaluation design and the planned use of collected data for Quality improvement purposes. A link to the online CC educational training was shared within the same email, embedded in a PowerPoint presentation titled ‘A guide to Cultural Competency training resource’ (supplementary materials), along with a self-assessment form discussed below. Informed consent to participate was assumed for all participants who chose to complete the training and self-assessment form.

### Educational training intervention

The CC training used in this study is the ‘Cultural Competence and Cultural Safety’ e-learning tool developed by Health Education England. This course comprises three sessions, each lasting 20–30 minutes (eLearning for healthcare, 2017). It aims to help clinicians understand how culture and health intersect and influence healthcare outcomes. Developed in collaboration with the Royal College of Midwives and other key stakeholders, the training is available for free to all healthcare professionals at https://www.e-lfh.org.uk/programmes/cultural-competence/.

The first two modules of the training course, titled ‘Introduction to Culture’ and ‘Cultural Competence’, were used in this project. The third module, which is specific to Maternity Services, was excluded as it was not relevant to participants who assess and treat patients with MSK conditions.

There was no capacity as part of this project to allow for dedicated time to undertake the training, so participants completed the training voluntarily during their personal time. On average, the training required approximately 1 hour to complete. Participants were also directed to optional additional learning resources available following completion of the programme should they wish to pursue further development.

### Measurements

The tool used to evaluate the effects of the training intervention was a 39-item Cultural Competency Self-Assessment (CCSA) form [supplementary materials]. Within this manuscript, the acronym CCSA refers exclusively to this newly developed form and is not used for the Cultural Competence Self‑Assessment Checklist, to avoid confusion.

The CCSA form was developed by adapting selected items from two existing tools: the Cultural Competence Self-Assessment Checklist (Central Vancouver Island Multicultural Society, 2022) and the Cultural Competency Assessment (CCA) tool (Khanna *et al*., 2009). Although informed by these sources, the CCSA form is a new and distinct instrument specifically tailored to align with the aims and objectives of this project.

The Central Vancouver Island Multicultural Society checklist is publicly available and explicitly allows adaptation and use. Similarly, the CCA tool developed by Khanna *et al*. (2009) was accessible through their published paper. Permission to use the CCA tool was obtained via an email from the lead author. The adaptation process involved carefully selecting relevant items from both sources and modifying them to create a comprehensive assessment tool tailored to the context of this study.

The CCSA form was organized into four sections: Demographic Information (6 items), Awareness Statements (5 items), Knowledge Statements (22 items), and Skills Statements (6 items). Responses in the awareness, knowledge, and skills categories were collected using a 5-point Likert scale. Demographic questions gathered data on participants’ gender, ethnicity, language competency, and profession. The remaining sections assessed changes in awareness, knowledge, and skills reported by participants after the training. Pre- and post-training responses focused on the three constructs of CC: awareness, knowledge, and skills. Each construct was scored based on its respective points, with a maximum of 25 points for awareness, 110 points for knowledge, and 30 points for skills, leading to a total possible score of 165 points.

### Data collection and analysis

Once participants finished the training, they were asked to complete two CCSA forms: one to evaluate their perceived CC after having completed the training and the second to capture their perception of their CC before undertaking the training. Unlike a traditional pre-then-post evaluation, where participants answer questions before and after the training, the post-then-pre method involves participants answering questions only after completing the intervention, with questions addressing their perceived status before and after the training (Khanna *et al*., 2009). This method is time-efficient, less intrusive for participants, and helps avoid pre-test sensitivity and response shift biases, which can result from over- or underestimation in pre-tests (Klatt and Taylor-Powell, 2005). It is particularly recommended for evaluating short-term training programmes (Pratt *et al*., 2000; Khanna *et al*., 2009).

Participants returned the completed CCSA forms via email to an assigned third person, who collected, collated, and anonymized the forms. Data were collected and tabulated in Excel for analysis.

Demographic data were analysed using descriptive statistics. To assess changes in perceived awareness, knowledge, and skills following the training, responses before (pre) and after (post) training were compared using SPSS v.28.0. Mean aggregate scores were calculated for each construct – awareness, knowledge, and skills – as well as for the overall CC score. Pre- and post-training mean scores were compared using a paired samples *t*-test to evaluate changes in participants’ perceived CC following the training.

Based on the findings, recommendations were made to improve future training interventions and further promote culturally competent patient care.

## Results

Seventeen out of 69 invited physiotherapists completed the training and returned the post-then-pre CCSA forms. Of these 17 participants, two were excluded from data analysis due to errors in form completion: one participant did not complete the post-training awareness section, and the other did not finish the entire pre-training part of the form. Table 1 provides the demographic details of participants.

**Table 1.** Demographic characteristics of all participants

Demographic characteristics		***n***
Gender		
Female		8
Male		5
Not disclosed		2
Self-identified ethnic/racial identity		
White/British		8
South Asian descent/African		1
White		3
Indian		1
Bilingual		
Yes		4
No		11
Profession		
Physiotherapist		15
No of years in profession		15
Minimum	1	
Maximum	30	
Mean	13.73	
Working as first contact practitioner		
Yes		5
No		10

### Cultural competency scores

The total cultural competency (CC) scores ranged from 101 to 128 on the pre-test and 112 to 161 on the post-test, with a maximum possible score of 165 points. The mean pre-test score was 117, while the mean post-test score was 135. The largest increase observed was from 128 to 161, and one participant experienced a slight decrease, with their score dropping from 113 to 112. All other participants showed an increase in their post-training scores.

**Figure 1.**
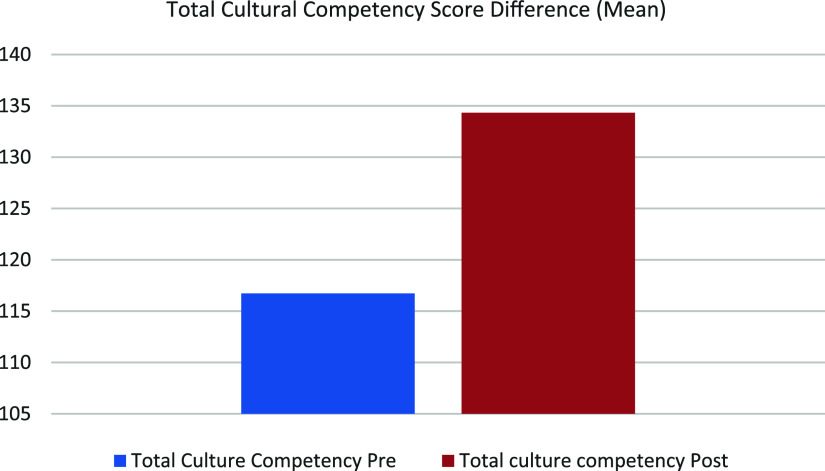
Mean scores for total cultural competency before and after the intervention.

Mean post-test scores (M = 134.33, SD = 12.93) were greater than mean pre-test scores (M = 116.73, SD = 9.46) (Table 2 and Figure 1). Overall, CC scores increased by an average of 17.60 points after training. This change was statistically significant, *t*(14) = –6.530, *p* < .001 (Table 3).

**Table 2.** Paired samples mean pre-post-test scores for cultural competency

Paired samples statistics					
		Mean	*N*	Std. deviation	Std. error mean
Pair 1	Total cultural competency pre	116.73	15	9.460	2.443
	Total cultural competency post	134.33	15	12.938	3.340

**Table 3.** Paired samples *t*-test for pre-post-test scores for cultural competency

Paired samples test										
		Paired differences					*t*	df	Significance	
					95% confidence interval of the difference					
		Mean	Std. deviation	Std. error mean	Lower	Upper			One-sided *p*	Two-sided *p*
Pair 1	Total cultural competency pre – Total cultural competency post	−17.600	10.439	2.695	−23.381	−11.819	−6.530	14	<.001	<.001

### Cultural awareness, knowledge, and skills

**Figure 2.**
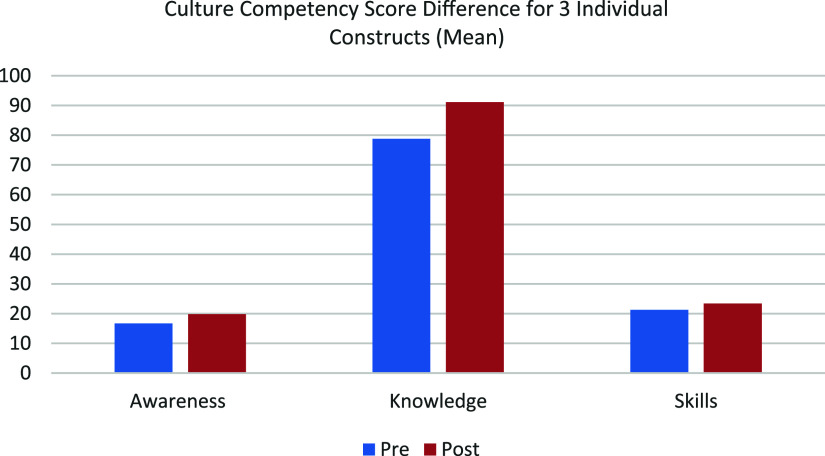
Mean scores for awareness, knowledge, and skills before and after the intervention.

The pre- and post-training mean scores for cultural awareness, knowledge, and skills were compared to assess changes following the training intervention, with results shown in Table 4 and Figure 2. Significant increases were observed across all three constructs, indicating meaningful improvements in participants’ perceived CC. Detailed statistical results are presented in Table 5.

**Table 4.** Paired samples mean pre-post-test scores for cultural awareness, knowledge, and skills

Paired samples statistics						
			Mean	*N*	Std. deviation	Std. error mean
Pair 1	Awareness total score	Pre	16.67	15	2.992	0.773
		Post	19.8	15	3.364	0.868
Pair 2	Knowledge total score	Pre	78.8	15	9.073	2.343
		Post	91.13	15	10.743	2.774
Pair 3	Skills total score	Pre	21.27	15	2.987	0.771
		Post	23.4	15	3.158	0.815

**Table 5.** Paired samples *t*-test of pre-post-test scores for cultural awareness, knowledge, and skills

Paired samples test										
		Paired differences					*t*	df	Significance	
		Mean	Std. deviation	Std. error mean	95% confidence interval of the difference				One-sided *p*	Two-sided *p*
					Lower	Upper				
Pair 1	Awareness pre total score – awareness post total score	−3.133	2.264	0.584	−4.387	−1.880	−5.361	14	<.001	<.001
Pair 2	Knowledge pre total score – knowledge post total score	−12.333	9.309	2.404	−17.489	−7.178	−5.131	14	<.001	<.001
Pair 3	Skills pre total score – Skills post total score	−2.133	1.807	0.467	−3.134	−1.132	−4.571	14	<.001	<.001

## Discussion

The aim of this project was to evaluate the impact of an online training intervention on perceived CC among community MSK physiotherapists caring for patients from diverse cultural and ethnic minority backgrounds. For the purpose of this project, CC was assessed using the following three constructs: cultural awareness, knowledge, and skills.

Results showed that a brief training programme effectively improved self-reported CC across all three constructs. The greatest improvement was seen in cultural knowledge, followed by awareness, and then skills. Knowledge scores increased by 12.33 points, awareness by 3 points, and skills by 2.13 points. Overall, the training resulted in short-term improvements in CC among physiotherapists, supporting the use of such brief training programmes to help prepare clinicians to better serve diverse populations.

The greater improvement in the knowledge section, compared to skills or awareness, was expected given the focus of the CC training programme. The programme strongly emphasized enhancing participants’ understanding of diverse cultural and ethnic groups through targeted educational content.

To enhance improvements in the awareness construct, CC training should educate participants about ethnocentrism and encourage them to reflect on their own beliefs, values, and biases. The aim is to help participants understand how cultural beliefs shape their conscious and unconscious thoughts, influence behaviours, and affect the therapeutic relationship (O’Shaughnessy and Tilki, 2007; Ingram, 2011; Bauer and Bai, 2015).

To improve the cultural skills construct, training should focus on enhancing cross-cultural communication skills and the ability to conduct effective intercultural consultations to address barriers to treatment adherence (Brathwaite, 2005; Alizadeh and Chavan, 2015). Rothlind *et al*. (2021), in their qualitative study, highlighted the use of a virtual patient system as a valuable tool for improving culturally relevant assessment skills and stimulating reflection. Participants can also develop new skills through readings, workshops, and collaboration with community leaders (Bauer and Bai, 2015; Slobodin *et al*., 2021).

The key finding from this project – that an educational intervention with pre- and post-tests can demonstrate an increase in CC – is consistent with studies by Khanna *et al*. (2009) and Fryer *et al*. (2020). Likewise, Sung and Park (2021) reported similar results using a mobile app-based CC training programme for nurses.

In this project, the online CC training averaged one hour in duration. Evidence from previous studies suggests that even brief training can enhance specific components of CC. Alizadeh and Chavan (2015), in their systematic review, identified cultural awareness, cultural knowledge, and cultural skills as the three core components of CC across various models. Supporting this, Delgado *et al*. (2013) found that a one-hour educational intervention significantly improved nurses’ cultural awareness. Similarly, Brathwaite (2005) showed that cultural knowledge improved through a series of educational sessions delivered over five consecutive weeks. Horky *et al*. (2017) further demonstrated a notable increase in self-reported CC skills following just two weeks of online training.

Systematic reviews by Jongen *et al*. (2018) and Vella *et al*. (2022) have highlighted significant variations in the educational content, delivery methods, and duration of CC training for healthcare professionals, making it difficult to determine its consistent impact on learning outcomes. Despite this variability, the evidence supports the effectiveness of targeted educational interventions in enhancing the core components of CC.

While this project demonstrated significant short-term improvements in physiotherapists’ cultural knowledge, awareness, and self-reported skills, such gains do not necessarily indicate a change in clinical practice or behaviours (Simons *et al*., 2018; Lee *et al*., 2025). Furthermore, research assessing the effect of CC training on patient outcomes remains limited, and there is no clear evidence of its impact on patient experiences or outcomes (Chae *et al*., 2020).

This project represents an important first step in a broader quality improvement pathway, and future projects should also evaluate the effectiveness of CC training on patient outcomes. Establishing a clear connection between CC training for health professionals and patient outcomes will require stronger study designs, consistent theoretical frameworks, and the use of validated outcome measures.

## Limitations

This project had several limitations that may have influenced the outcomes, including a small sample size, variations in participant motivation, the short duration of the project, and the lack of validity and reliability testing for the CCSA form used.

First, the small sample size limits the statistical power and generalizability of the findings. However, the results were still significant in showing an increase in CC within this specific group of MSK physiotherapists.

Second, the varying levels of professional and personal motivation to engage with the training were a challenge. Participants’ desire to improve their CC may have affected their commitment to the programme and contributed to a lower response rate. Additionally, since the training occurred during the participants’ personal time, this could have impacted their motivation to fully engage with the content.

Third, the short time frame of the project limited the development of a more structured CC training programme. The lack of time prevented the use of established frameworks such as the Campinha-Bacote Model of Cultural Competency in healthcare delivery (Campinha-Bacote, 2002), which could have provided a more comprehensive foundation for the training. Future projects might benefit from incorporating such models to ensure a more systematic and sustained approach to enhancing CC.

Lastly, the validity and reliability of the CCSA form used to measure the improvements were not tested in this project. Future studies should ensure that the assessment tools used to measure CC undergo proper psychometric evaluation to ensure their accuracy and reliability.

## Conclusion

As the patient population in the United Kingdom becomes increasingly diverse, healthcare providers must navigate varying cultures, values, and beliefs to ensure equitable care. Patients from diverse cultural and ethnic backgrounds are particularly vulnerable to health inequalities. This project demonstrated that CC can be improved through targeted training, with participants showing enhanced awareness, knowledge, and skills in providing culturally competent care. However, CC is a lifelong, evolving process that requires continuous education and practice.

To maintain these improvements, additional PDSA cycles are recommended, with refined educational sessions adapted to specific cultural or migrant groups and targeted training focused on individual CC components. Increasing the frequency and depth of these sessions will better address the complexities of CC. Future projects should also evaluate the effectiveness of CC training on patient outcomes.

Lastly, it is essential to broaden the scope of CC to address issues beyond racial and ethnic differences. Incorporating factors such as sexual orientation, socio-economic status, disability, neurodiversity, and the type and timing of care is crucial for reducing health inequalities and improving the overall quality of patient care.

## Supporting information

Gohil et al. supplementary material 1 Gohil et al. supplementary material

Gohil et al. supplementary material 2 Gohil et al. supplementary material

Gohil et al. supplementary material 3 Gohil et al. supplementary material

Gohil et al. supplementary material 4 Gohil et al. supplementary material

## Supplementary material

The supplementary material for this article can be found at https://doi.org/10.1017/S1463423625100765.
