# Supplementary material for: Mind the (cultural competency) gap: bridging cultural barriers in physiotherapy practice – a quality improvement project
Source: Prim Health Care Res Dev. 2026 Feb 2;27:e14. doi: 10.1017/S1463423625100765 (PMC12931987; doi:10.1017/S1463423625100765)
Supplement: Gohil et al. supplementary material 1 — Gohil et al. supplementary material [file S1463423625100765sup001.pdf]

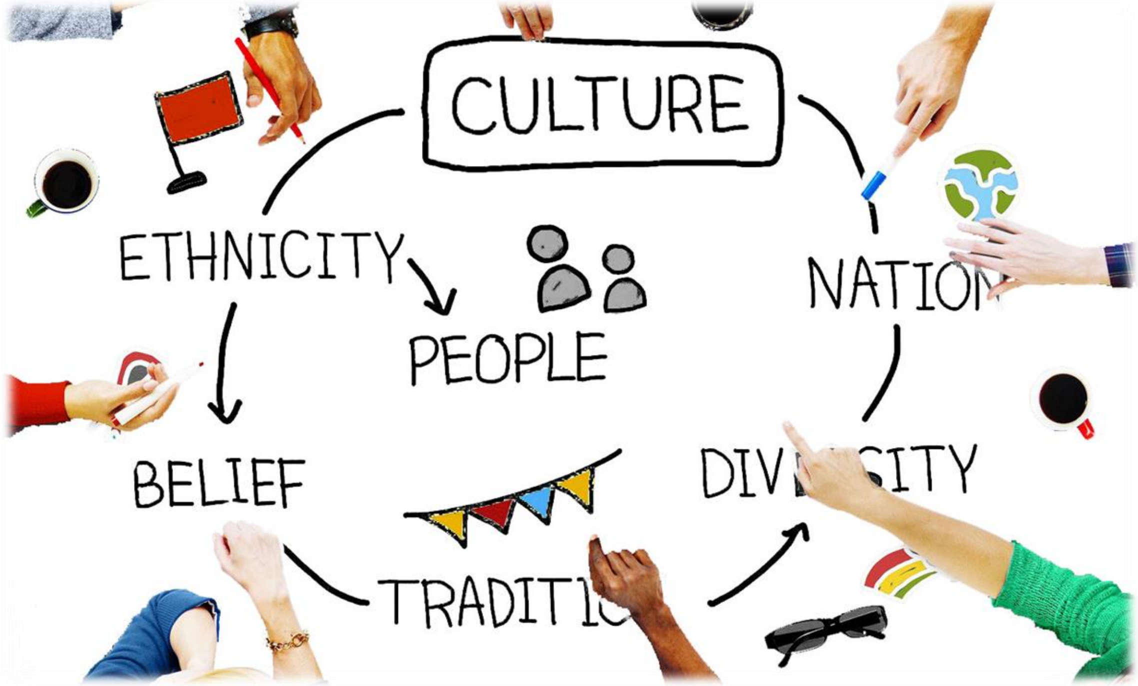

A Guide To Culture Competency Training Resource

Manish Gohil- MSK/FCP Physiotherapist

m.gohil1@nhs.net

Why This Training?

For Quality Improvement project (QIP)

This QIP is part of my PG Certificate First Contact Practitioner Course.

Project Information

Title: Improving levels of Cultural Competency among physiotherapist caring for patients from diverse cultural and ethnic minority backgrounds.

Research question: Does cultural competency training produce a measurable change in the self-assessment of participant’s awareness, knowledge and skills related to the care of patients from diverse cultural and ethnic backgrounds?

Need For Culture Competency ?

In the United Kingdom, as demonstrated in other countries, the growth of various ethnic communities and linguistic groups, each with their own cultural traits and health profiles, presents a complex challenge to healthcare practitioners and policy makers in terms of achieving equitable access to healthcare.

To address health inequalities, providers must develop culturally competent practice that is mindful of the values, beliefs and practices of every patient.

Training in ‘cultural competence’ has originated as a response to managing the complexity of a culturally diverse UK patient population.

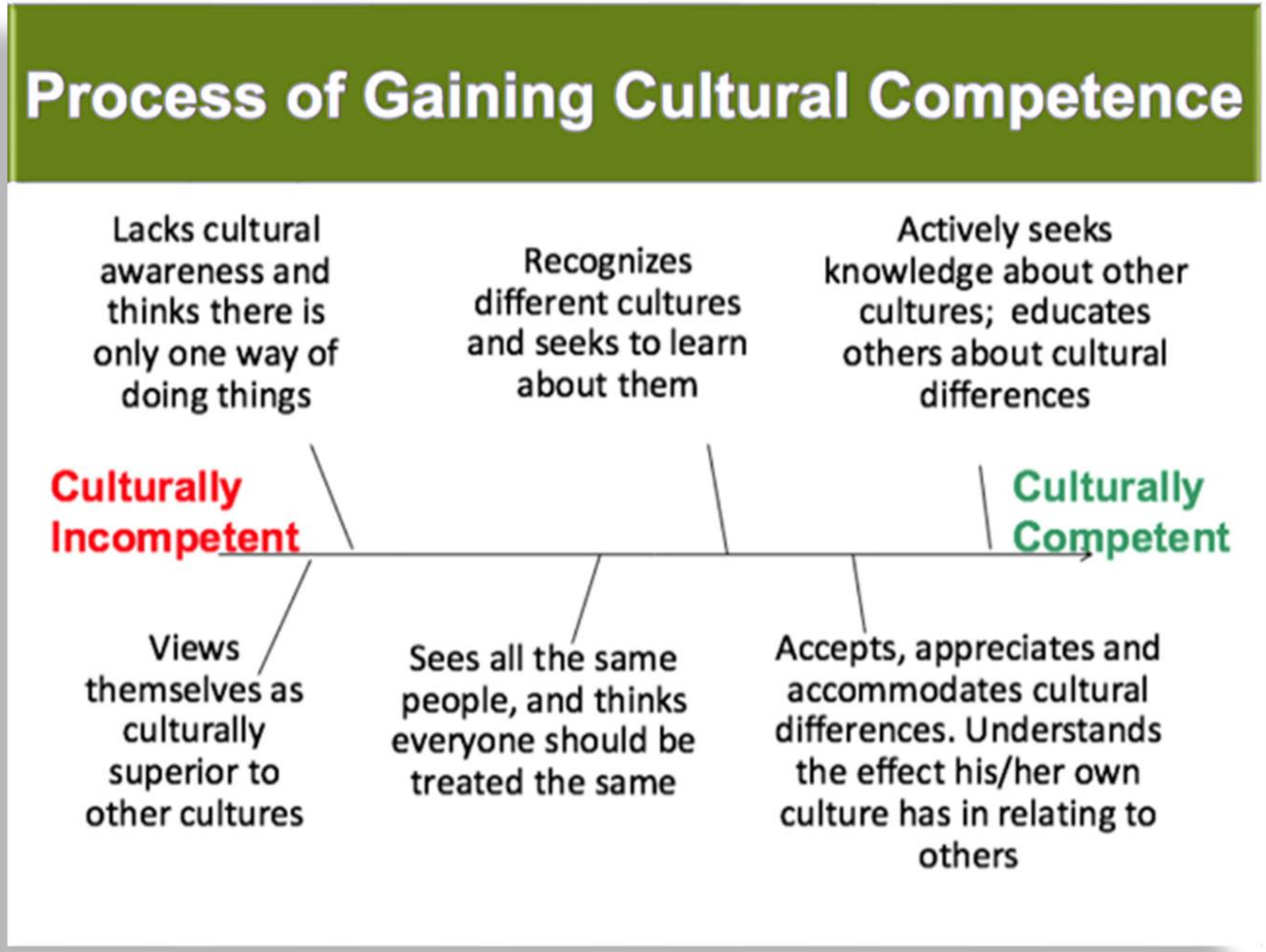
Process of Gaining Cultural Competence
Lacks cultural awareness and thinks there is only one way of doing things
Recognizes different cultures and seeks to learn about them
Actively seeks knowledge about other cultures; educates others about cultural differences
Culturally Incompetent
Culturally Competent
Views themselves as culturally superior to other cultures
Sees all the same people, and thinks everyone should be treated the same
Accepts, appreciates and accommodates cultural differences. Understands the effect his/her own culture has in relating to others

Resource1: Cultural Competence eLearning

(click below link)

[Cultural Competence - elearning for healthcare (e-lfh.org.uk)]

It includes 3 sessions:

- Introduction to Culture (20 mins- Essential)
- Cultural Competence (20 mins- Essential)
- Cultural Competence and Maternity Services (Optional session)

Introduction to Culture (20 mins session)

Having completed this session you will be able to:

- Explain why 'culture' is important to health professionals in modern Britain
- Describe what constitutes culture
- Describe how culture can impact individuals' values, beliefs, behaviours and decision-making
- Explain how culture can impact care and treatment decisions
- Describe the difference between cultural norms and harmful traditional practices

Cultural Competence (20 mins Session)

Having completed this session you will be able to:

- Describe the key components of a culturally competent service
- Describe what being culturally competent in your area of work means
- Explain why health professionals have a responsibility to develop an awareness of diverse cultures
- Explain the importance of self-awareness and the impact your own prejudices, beliefs, values and cultural roots have on your interactions and care of patients who are not from your own social and cultural group
- Describe the impact of making assumptions based on individuals' cultural background or behaviour in particular circumstances
- Explain what is meant by the term 'cultural safety'

Resource2: Culture and Pain Talk

- Culture and Pain talk by Mohammad Shoiab- Virtual in-service training organised by Leeds community NHS Trust on 13th October 2022
- H:\MSK Physiotherapy\Training, Education & Development\IST repository of past presentations\Oct 2022 - Culture & Pain; Biopsychosocial screening & assessment\Culture & Pain slides - 14-10-2022 Leeds.pdf

Adobe Acrobat Document

- H:\MSK Physiotherapy\Training, Education & Development\IST repository of past presentations\Oct 2022 - Culture & Pain; Biopsychosocial screening & assessment\Leeds Community MSK Service Training Morning-20221013_083220-Meeting Recording.mp4

Additional Resource: Research Papers for further reading!!

Adobe Acrobat Document

Adobe Acrobat Document

Adobe Acrobat Document

Culture Competency Self-Assessment (CCSA) Form

Microsoft Word Document

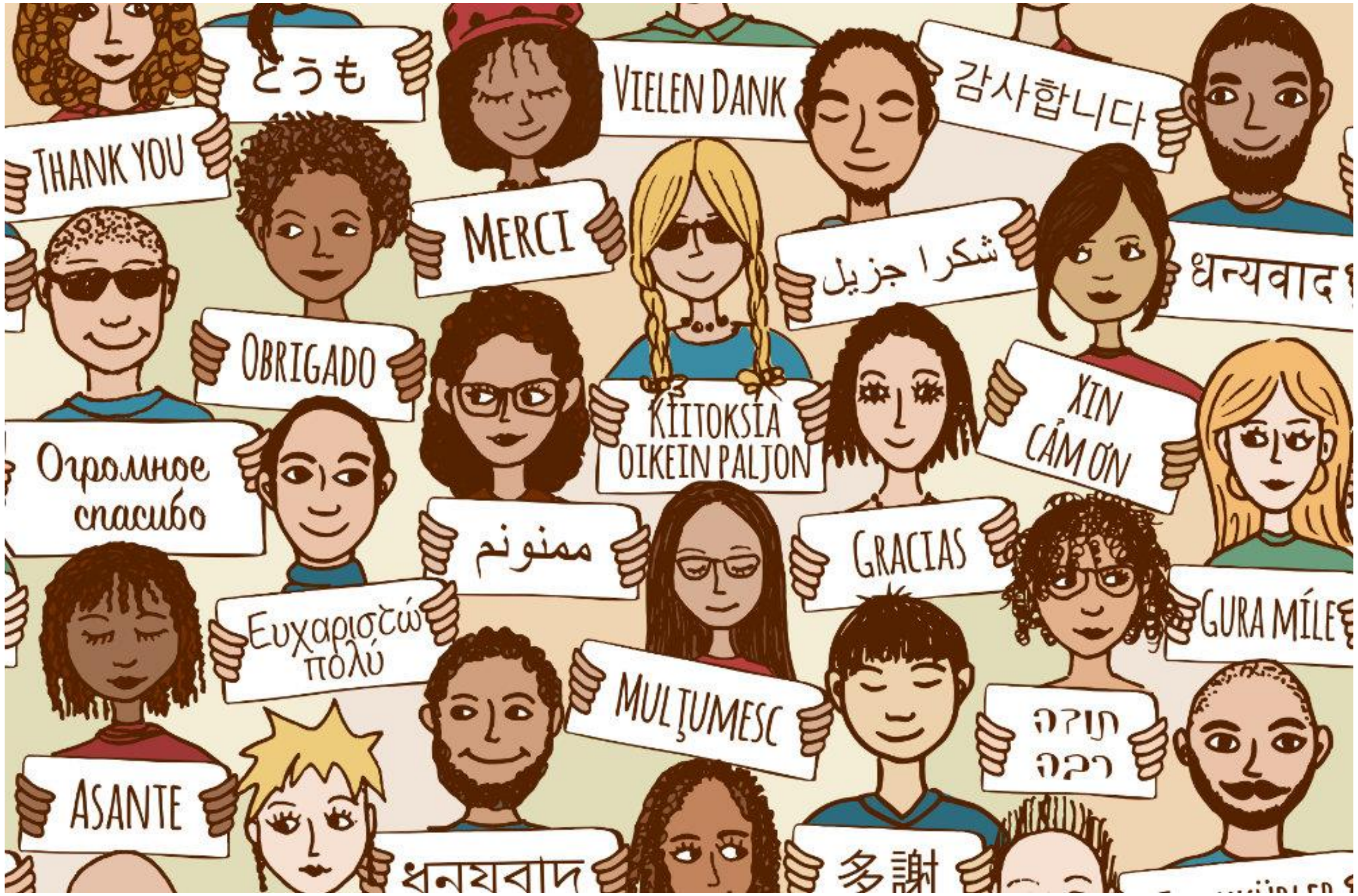

とうも
VIELEN DANK
감사합니다
THANK YOU
MERCI
شكرا جزيل
धन्यवाद
OBRIGADO
KIITOKSIA OIKEIN PALJON
XIN CẢM ƠN
Огромное спасибо
ممنونم
GRACIAS
GURA MÍLE
Ευχαριστώ πολύ
MULŢUMESC
תודה רבה
ASANTE
多謝
