# Supplementary material for: Mind the (cultural competency) gap: bridging cultural barriers in physiotherapy practice – a quality improvement project
Source: Prim Health Care Res Dev. 2026 Feb 2;27:e14. doi: 10.1017/S1463423625100765 (PMC12931987; doi:10.1017/S1463423625100765)
Supplement: Gohil et al. supplementary material 3 — Gohil et al. supplementary material [file S1463423625100765sup003.pdf]

Culture Competency Self-Assessment (CCSA) Form

Part 1: Demographic Information

Gender:

Self-identified ethnic/racial identity:

Are you bilingual /Do you speak any languages other than English? Yes /No
If yes, please specify _________________________

Profession:
Years in profession:

Working as First contact practitioner (FCP): Yes/No

Part 2: Awareness											
Pre-training						Please mark (x) to your responses	Post-training				
	Never	Rarely	Somewhat often/ occasionally	Very often/well	Always/very well		Never	Rarely	Somewhat often/ occasionally	Very often/well	Always/very well
	1	2	3	4	5		1	2	3	4	5
1						I am aware of my own cultural values, beliefs, bias, and opinions of other cultures.					
2						I am aware of my discomfort when I encounter differences in race, colour, religion, sexual orientation, language, and ethnicity.					
3						I am aware of my stereotypes as they arise and have developed personal strategies for reducing the harm they cause.					
4						I am aware of how my cultural perspective influences my judgement about what are ‘appropriate’, ‘normal’, or ‘admirable’ behaviours, values, and communication styles.					
5						I am aware of possible barriers or biases in my beliefs, values, and perceptions on others that may create disparities or negative outcomes for patients from diverse cultural and ethnic minority backgrounds					

Part 3: Knowledge											
	Never	Rarely	Somewhat often/ occasionally	Very often/well	Always/very well	Please mark (x) to your responses	Never	Rarely	Somewhat often/ occasionally	Very often/well	Always/very well
	1	2	3	4	5		1	2	3	4	5
1						I know that differences in colour, culture, ethnicity etc. are important parts of an individual's identity which they value and so do I. I will not hide behind the claim of "colour blindness".					
2						I recognize that cultures change over time and can vary from person to person, as does attachment to culture.					
3						Health care systems and providers discriminates against patients based on:					
						a. How well they speak English					
						b. Their race or ethnic background					
						c. Their sexual orientation					
						d. Physical disability					
4						Culture, race, and ethnicity can be interchangeably used.					
5						Health differences exist between races/ethnic groups					
6						Patients' health beliefs impact access to health care					
7						Patients' health beliefs impact use of health care					
8						Patients' race/ethnicity influences access to health care					
9						Cultural factors influence communication between a patient and a provider					
10						Minority patients experience racism and discrimination while seeking health care					
11						Health care providers should:					
						a. Learn about the cultural and health beliefs of patients					
						b. Greet patients in a culturally appropriate manner					
						c. Ask patients about their perspectives on illness					
						d. Ask patients about the use of folk remedies					
						e. Examine patients in a culturally appropriate manner					
						f. Develop a culturally sensitive health plan for patients					
						g. Acknowledge family members' roles in patients' health care decisions					
						h. Pay attention to nonverbal cues or gestures when interacting with patients					
12						I recognize that achieving cultural competence involves a commitment to learning over a lifetime.					

Part 4: Skills											
	Never	Rarely	Somewhat often/ occasionally	Very often/well	Always/very well	Please mark (x) to your responses	Never	Rarely	Somewhat often/ occasionally	Very often/well	Always/very well
	1	2	3	4	5		1	2	3	4	5
1						I can adapt my communication style to effectively interact with people who speak in ways that are different from my own					
2						I can effectively work with health care interpreters					
3						I can successfully deal with cross-cultural adherence problems					
4						I can successfully deal with cross-cultural misunderstandings					
5						I can effectively deal with patients with limited English proficiency					
6						I know it is important to pay attention to cultural expressions of pain, distress, isolation, & disagreement					
